# Enhanced neural phase locking through audio-tactile stimulation

**DOI:** 10.3389/fnins.2024.1425398

**Published:** 2024-10-02

**Authors:** Mels Jagt, Francesco Ganis, Stefania Serafin

**Affiliations:** ^1^Multisensory Experience Lab, Department of Architecture, Design and Media Technology, Aalborg University Copenhagen, Copenhagen, Denmark; ^2^Life Sciences Engineering (Neuroscience and Neuroengineering), École Polytechnique Fédérale de Lausanne (EPFL), Lausanne, Switzerland

**Keywords:** frequency following response, phase locking, fundamental frequency, pitch, speech, audio-tactile, multisensory, vibrotactile

## Abstract

Numerous studies have underscored the close relationship between the auditory and vibrotactile modality. For instance, in the peripheral structures of both modalities, afferent nerve fibers synchronize their activity to the external sensory stimulus, thereby providing a temporal code linked to pitch processing. The Frequency Following Response is a neurological measure that captures this phase locking activity in response to auditory stimuli. In our study, we investigated whether this neural signal is influenced by the simultaneous presentation of a vibrotactile stimulus. Accordingly, our findings revealed a significant increase in phase locking to the fundamental frequency of a speech stimulus, while no such effects were observed at harmonic frequencies. Since phase locking to the fundamental frequency has been associated with pitch perceptual capabilities, our results suggests that audio-tactile stimulation might improve pitch perception in human subjects.

## 1 Introduction

The combination of both psychophysical and neurophysiological methodologies have aimed to provide a comprehensive understanding of how physical stimuli are transformed into perceptual experiences through neural processes. This paper focuses on the perception of pitch. Initially, we will outline how pitch is manifested within the auditory system, followed by a discussion on its analogous representation via vibrotactile stimulation. Subsequently, existing interactions between auditory and vibrotactile pitch processing are explored, establishing a solid foundation prior to introducing our research approach, which is presented in the end of this section.

**Figure 1:**
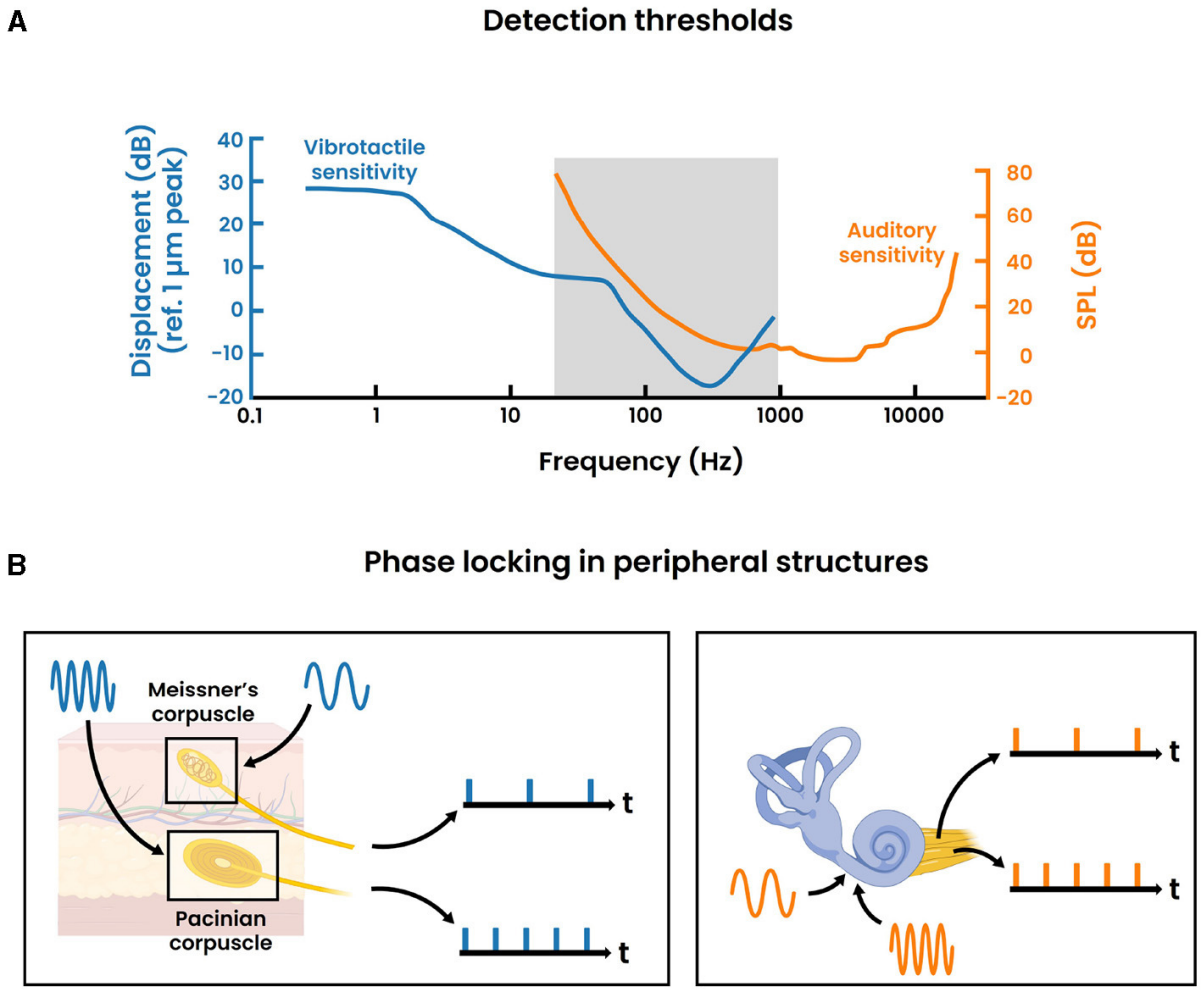
Parallels between the auditory and vibrotactile modality. **(A)** Detection thresholds for the auditory and vibrotactile modality across different frequencies. The vibrotactile sensitiviy curve is derived from Gescheider et al. (2002) where 700 ms sinusoidal stimuli are presented through a 0.72 cm^2^ circular contactor at the index fingertip of the right hand. The auditory sensitivity curve is based on the International Standard ISO 389-7: 2003 for pure tones under free-field and diffuse-field listening conditions. The gray area indicates the frequency range where both curves overlap. **(B)** Phase locking activity in the peripheral structures of the vibrotactile modality (left) and auditory modality (right) for arbitrary stimulus frequencies respectively. Single nerve fibers are displayed for illustrative reasons. In reality, the precise synchronization should be regarded as the response of a population of nerve fibers.

The psychophysical study of pitch perception has a long history, stretching back to the period where Pythagoras investigated the connection between the length of a plucked string and its excitation frequency. Meanwhile it is understood that sound essentially involves airborne pressure waves that are commonly characterized by three physical attributes: frequency, amplitude and time/phase (Yost, 2009; Merchel and Altinsoy, 2020). Pitch, the degree to which a sound is perceived as “high” or “low”, is arguably the most critical perceptual feature of sound. This attribute plays an essential role in musical appraisal, speech and the identification of sources (Kraus, 2021). In the case of simple pure tones representing sinusoidal waves with a singular frequency, pitch directly correlates to its frequency (Yost, 2009). The human auditory system is capable of detecting these pure tones across a frequency spectrum from 20 Hz to 20,000 Hz, albeit with varying degrees of sensitivity across this range. As illustrated in Figure 1A, the hearing has lowest detection thresholds to frequencies between approximately 300 Hz–7,000 Hz. For more complex stimuli, the perception of pitch is not always as straightforward as with pure tones. Many everyday sounds involve harmonic complexes which are characterized by a fundamental frequency, the first harmonic, alongside additional frequency components that occur at integer multiples of this fundamental frequency, known as higher harmonics. Generally, the pitch can be directly derived from the fundamental frequency, however this is not always the case as illustrated with the missing-fundamental stimulus (Vogt and Kasper, 2023). A more elaborate discussion on the different conditions that lead to pitch perception can be found in Yost (2009).

**Figure 2:**
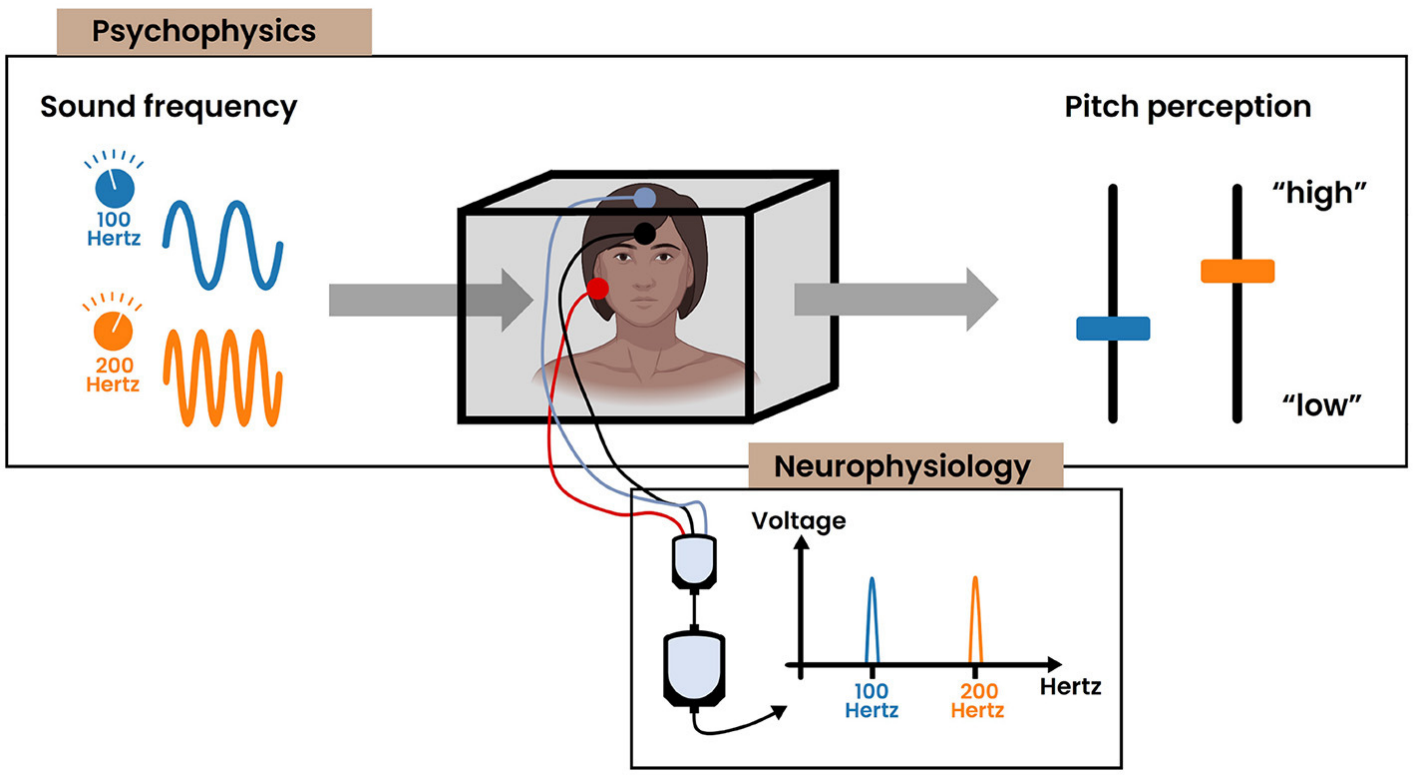
The common strategy for exploring the relationship between sensation and perception, combining the results obtained by the field of psychophysics and neurophysiology. The subject of study is displayed centrally in a box. **(Psychophysics)** Illustration depicting the positive correlation between the physical frequency of a sinusoidal stimulus and the resultant pitch perception. **(Neurophysiology)** Examining the neural mechanisms that underlie these psychophysical observations. In this example, the frequency-following response (FFR) captures the phase locking activity in the brain stem to the frequency of the auditory stimulus.

Additionally, over the past century, neurophysiological research into pitch perception has progressed our understanding of the underlying neural mechanisms. Today, it is commonly believed that much of the pitch information is contained within the temporal code of spiking neurons (Plack et al., 2014). This is well represented in the auditory periphery where the auditory nerve fibers synchronize their firing (i.e., phase lock) to the frequency of the vibration at each place on the basilar membrane, which is sensed through mechanoreceptors (i.e., the inner hair cells) in the cochlea (Figure 1B). The upper limit of this phase locking behavior is about 5 kHz, though the exact number is still under debate (Verschooten et al., 2019). Subsequently, pitch could be encoded by synchronization to either the stimulus' temporal fine structure (≤ 5 kHz) or the temporal envelope. Toward higher stages in the ascending auditory pathway, the upper limit of phase locking progressively decreases. The frequency-following response (FFR) is a non-invasive electrophysiological measure that captures phase locking activity (≤ 2 kHz) within the brain stem (Kraus et al., 2017) (Figure 2). Commonly, a “vertical” one-channel electrode configuration is used with the active electrode on the top head (Cz), the reference electrode on the earlobe (A1/A2) and the ground electrode on the forehead (Fpz). Although this configuration primarily targets the sum of synchronized neural activity in the inferior colliculus, it is believed that the FFR in reality represents multiple neural sources that all operate in concert with each other (Coffey et al., 2019). Interestingly, differences in FFR strength (i.e., phase locking efficacy to the fundamental frequency) has been correlated with differences in pitch perception. For example, the work of Krishnan et al. has demonstrated that tone language speakers (e.g., Chinese, Thai) have an enhanced FFR compared to non-tone language speakers (e.g., English) (Krishnan et al., 2005, 2010). Additionally, improved pitch discrimination following short-term training has been shown to strengthen the FFR (Carcagno and Plack, 2011). These results suggest that the FFR likely bears pitch relevant information, despite not directly depicting the precise mechanism of pitch extraction. Moreover, besides pitch, it has to be emphasized that the FFR also contains information relevant to other aspects of auditory processing. An extensive overview of the latter is presented in Krizman and Kraus (2019). Ascending further toward the cortex, the phase locking ability dimishes drastically. As a consequence, the temporal code for pitch is likely transformed into another type of encoding. Less is understood about these higher order representations, while current research mainly focuses on finding cortical “pitch centers” using different techniques (e.g., fMRI, EEG) (Plack et al., 2014).

To this point, the discussion on pitch perception has only focused on its manifestation within the auditory system. However, a comparable phenomenon exist for the somatosensory system. More precisely, it has been demonstrated that vibrotactile stimuli, referring to the detection of vibrations on the skin, can also be perceived as “high” or “low”, akin to auditory stimuli (Prsa et al., 2021). A number of psychophysical analogies can be drawn between these two modalities (Merchel and Altinsoy, 2020). That is, most physical properties of airborne vibrations (e.g., frequency, amplitude) find their direct counterpart in skin vibrations (Von Bksy, 1959). A significant distinction, however, is that vibrotactile stimuli can be perceived at different locations on the body. Here, regions with high (vibro)tactile acuity (i.e., hands or fingertips) have been characterized by a dense concentration of sensory receptors (Bruns et al., 2014; Purves, 2018). Many research efforts have been focused on the fingertip, and evidence seems to indicate that the perception of vibrotactile pitch for simple sinusoids also greatly depends on the vibration's frequency, while possibly being modulated by its amplitude (Prsa et al., 2021). Moreover, similar to the auditory system, a V-shaped sensitivity curve has been computed for sinusoidal skin vibrations (Figure 1A). In comparison, the frequency discrimination of vibrotactile stimuli exhibits a significantly lower resolution, with Weber fractions ranging from 0.2-0.3 in contrast to 0.003 as shown for the auditory system (Saal et al., 2016). Furthermore, while the frequency spectrum is somewhat restricted to a maximum of 1000 Hz, it is perceptually classified into two distinct categories: a 'flutter' range up to 50 Hz and 'smooth vibrations' for frequencies above 50 Hz, mirroring the auditory system. In both senses, the flutter range is characterized by the absence of pitch where individual cycles are discernible, oppposed to higher frequencies that generate the percept of a continuous signal with an identifiable pitch. Early work by Mountcastle et al. has linked these perceptual findings with neurophysiological studies (Mountcastle et al., 1969, 1972). Accordingly, it has been shown that primarily two types of mechanoreceptors are involved in the detection of vibrations within the somatosensory periphery. Specifically, the Meissner's corpuscles mainly account for the detection of the flutter range vibrations, while the Pacinian corpuscles are predominantly involved in the detection of the smooth vibrations. Intriguingly, these peripheral structures exhibit phase locking behavior analogous to that observed in the auditory periphery (Figure 1B). That is, the activity of afferent nerve fibers innervating these mechanoreceptors periodically entrain to the frequency of the vibrating stimulus, enabling synchronization to skin vibrations across the entire spectrum up to 1,000 Hz (Saal et al., 2016). Hence, this temporal code also carries stimulus information, and therefore potentially bears relevant information to vibrotactile pitch processing.

Besides these psychophysical and neurophysiological parallels in pitch perception across both sensory modalities, there is another empirical argument that further supports the existence of a close relationship. Particularly in the music domain, the sensation of sound can be coupled with vibrations on the skin. This becomes apparent in the setting of a live concert where vibrotactile stimuli complement auditory cues to enhance the musical journey for both the audience as well as the performers (Merchel and Altinsoy, 2018). Collectively, these observations motivate the investigation into the possible interactions between the auditory and vibrotactile (i.e., audio-tactile) modality, especially with respect to pitch processing. This view is consistent with the recent trend of the last few decades where the field of sensory processing has shifted from scientific research investigating each sensory modality in isolation, to the perspective of a highly interconnected, interactive multisensory network (Stein et al., 2014).

Over the past two decades, a considerable body of research has been dedicated to investigating the impact of audio-tactile interactions on human perception. Consequently, it has been shown that audio-tactile stimulation improves reaction speed (Sperdin, 2009), stimulus detection (Gillmeister and Eimer, 2007) and enhances the perceived loudness (Schrmann et al., 2004; Gillmeister and Eimer, 2007). Interestingly, these interactions seem to depend on the relative frequencies between both modalities (Wilson et al., 2010b,a), with the most pronounced effects observed when the frequencies of auditory and vibrotactile stimuli closely overlap. This observation also extends to audio-tactile influences on pitch perception. In subsequent sections, we primarily concentrate on the examination of audio-tactile interactions within the supra-flutter range of 50 Hz to 1000 Hz because: i) it contains a distinct and identifiable perception of pitch; ii) it represents the overlapping region where frequencies are perceptible to both the auditory and somatosensory systems. Accordingly, the work of Yau et al. has revealed that auditory and vibrotactile stimuli reciprocally bias each other, demonstrating that the concurrent presentation of a vibrotactile distractor significantly influenced the perception of auditory pitch, and vice versa (Yau et al., 2009, 2010). Concretely, using simple sinusoids, the pitch frequency of a stimulus in one modality was pulled toward the frequency of the distractor modality. Subsequent research following a crossmodal adaptation design further substantiated these observations, demonstrating how vibrotactile pitch perception is influenced by auditory stimuli using band pass noise (Crommett et al., 2017) and sinusoidal sweeps (Crommett et al., 2019) respectively. A key finding was that these results were only obtained when the frequencies of both modalities were sufficiently aligned. Collectively, this further implicates an intimate relationship between both modalities, and suggests shared interactive neural mechanisms regarding frequency processing.

Subsequent research efforts have been devoted to elucidate such neural foundations that facilitate these observed audio-tactile effects. In this regard, a significant number of studies in human subjects have utilized the fMRI neuroimaging technique, while specifically targeting the cerebral cortex. Accordingly, it has been shown that areas traditionally associated with a single sensory modality are susceptible to crossmodal influences. That is, auditory stimulation revealed robust and frequency-specific responses within the traditionally defined somatosensory regions of the parietal lobe (Prez-Bellido et al., 2018). In the same way, vibrotactile stimuli has shown to activate areas typically associated with auditory processing within the temporal lobe (Schrmann et al., 2006; Nordmark et al., 2012). Regarding the latter, it was specifically shown that 100 Hz sinusoidal vibrations selectively impacted the left auditory cortex, an area thought to have a specialized role in detecting fundamental frequencies (Nordmark et al., 2012). Consistent with these results, a more recent study revealed similar overlapping activation regions and highlighted their involvement in frequency specific processing (Rahman et al., 2020). While these results tentatively indicate the existence of shared neural populations regarding frequency processing, the direct link of these observations to the psychophysical audio-tactile interactions of pitch perception remains rather obscure. This challenge can be attributed in part to the inherent constraints of fMRI. While fMRI has proven effective for spatially identifying brain regions engaged in frequency processing, its relatively limited temporal resolution and the sluggishness of the blood oxygenation level dependent (BOLD) signal complicate the task of directly linking perceptual outcomes with underlying neural processing activities. Moreover, emerging evidence from animal studies has revealed extensive audio-tactile interactions within subcortical regions, involving both ascending and descending projections (Lohse et al., 2022). Consequently, the activities observed in cortical regions might merely reflect the crossmodal influences originating from these lower-level neural structures. This observation would not come completely unexpected, considering the analogous temporal coding mechanisms present in subcortical regions across both modalities.

To summarize, the pronounced similarities between the auditory and vibrotactile modalities concerning pitch processing, in combination with substantial perceptual evidence of audio-tactile interactions, collectively suggest the existence of shared neural pathways for frequency processing. Efforts to investigate such putative networks in human subjects, particularly focusing on the cerebral cortex, have yet to yield compelling evidence. Meanwhile, research in animal models indicates the presence of significant audio-tactile interactions at subcortical stages. Hence, it may be beneficial to explore analogous regions in human participants. Accordingly, the principal aim of this study is to address the latter proposition by exploring the following questions: i) is it possible to furnish evidence supporting the presence of audio-tactile interactions within subcortical structures in human subjects?; ii) should such evidence emerge, what would be its implications for the temporal coding mechanism? Our hypothesis states that vibrotactile stimuli complement auditory stimuli and improve phase locking acuity. Considering the efficacy of FFR in capturing subcortical phase locking activity to auditory stimuli, our research seeks to build upon this by incorporating a concurrent vibrotactile stimulus. Accordingly, this enables the investigation of how the synchronized neural activity in the brain stem is potentially modulated by the vibrotactile modality.

## 2 Materials and methods

Previous studies have predominantly utilized basic and coarse audio-tactile stimuli, such as simple sinusoidal waves, often delivered through insert earphones and applied to a single finger digit. This study intends to adopt a more natural stimulation paradigm. To achieve this, we choose to utilize real-world speech stimuli, presented through insert earphones, alongside an ergonomic vibrotactile controller that stimulates the entire hand. Most settings concerning the auditory stimulation and FFR recording are directly derived from Krizman et al. (2019). Details are presented below.

### 2.1 Participants

The dataset consisted of FFRs recorded from 22 healthy young adults (age: 28 ± 6) of which 11 are female. None of the participants had a history of neurological dysfunction or a reported hearing loss and all gave written consent to participate on voluntary basis.

### 2.2 Stimulus selection

The selected stimulus was identical for both the auditory and vibrotactile modality, and involved the 40-ms */da/* speech syllable. This */da/* is a generated speech sound (Klatt, 1980) with five formants. The syllable is characterized by an initial noise burst followed by a formant transition between the consonant and the vowel. More specifically, during the 40-ms, the fundamental frequency (F0) and the first three formants (F1, F2, F3) shift linearly (F0: 103–125 Hz, F1: 220–720 Hz, F2: 1,700–1,240 Hz, F3: 2,580–2,599 Hz). The formants F4 (3,600 Hz) and F5 (4,500 Hz) however remain constant.

**Figure 3:**
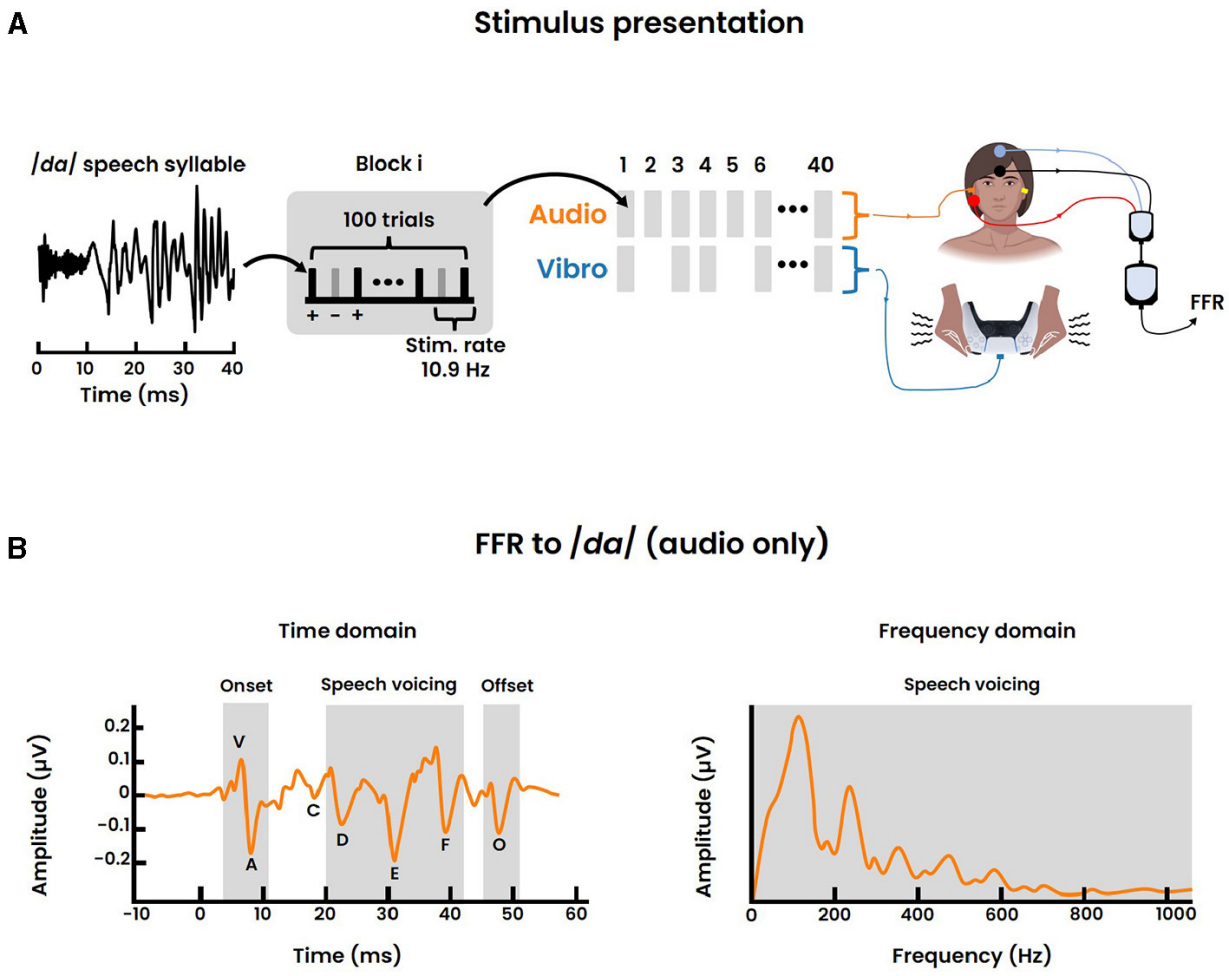
FFR recording. **(A)** Stimulus presentation of the 40-ms /*da*/ speech syllable for a single session. Each block consisted of 100 individual /*da*/ trials, presented in alternating polarity (+, -) with a stimulus rate of 10.9 Hz. In total 40 blocks were delivered with the randomized inclusion of the vibrotactile modality. Accordingly the FFR was recorded for both the audio and audio-tactile condition following a vertical electrode montage. **(B)** Normative FFR data to a 40-ms /*da*/ sound stimulus, adapted from Skoe et al. (2015). The time domain signal on the left displays the seven stereotypical peaks, representing: the stimulus onset response (“V”, “A”), the transition from stimulus onset to voicing onset (“C”), the voicing of the speech (“D”, “E”, “F”) and the offset response (“O”) respectively. The right graph displays the frequency domain of the speech voicing region of the FFR (19.5–44.2 ms). The largest peak in the spectrum represents the fundamental frequency.

This specific stimulus was chosen for the following reasons. First, */da/* is a relatively universal syllable found in the phonetic inventories of most European languages (Maddieson, 1984). Second, conventional FFR studies focusing on the auditory modality only, showed that this sound stimulus elicits a clear and replicable response (Figure 3B), thereby providing a normative database (Skoe et al., 2015). Last, the fundamental frequency, as well as the first formant, of /*da*/ lie well within the perceivable frequency range of the vibrotactile modality and are below subcortical phase locking limits.

### 2.3 Stimulus presentation

Vibrotactile stimulation was provided through a DualSense Controller (Sony Corporation, Tokyo, Japan) via USB connection. The reason for choosing this device is two-fold. First, the controller has an ergonomic casing that is rigorously tested to provide comfortable vibrations to a broad audience. Second, on both the left and right hand side, it has two built-in voice-coil actuators (ref. 622008, Foster Electric Company, Tokyo, Japan). These type of actuators are frequently employed for transmitting musical information via vibrotactile stimuli (Remache-Vinueza et al., 2021). For example, audio signals can be utilized to drive them with minimal or no further signal processing required, where pitch and loudness directly maps to the frequency and amplitude of the vibration respectively (Petry et al., 2018). Corollary, it is posited that such actuators are also adequate for conveying speech stimuli, including the /*da*/ used in this study. Nonetheless, given that the DualSense controller is primarily designed for entertainment purposes, further validation was necessary to evaluate its appropriateness for this research (see section 2.6). After the successful preliminary validation, vibrotactile signals were delivered bimanually and calibrated at 0.85 m/s^2^ peak-to-peak.

Auditory stimuli were presented through an IP30 insert earphone (RadioEar, Middelfart, Denmark) via the Steinberg UR44C audio interface (Steinberg Media Technologies GmbH, Hamburg, Germany). These earphones are electromagnetically shielded in order to minimize the stimulus artifact (i.e., the electrical signal produced by a transducer that could contaminate the FFR recording). Subsequently, auditory signals were delivered monoaurally in the right ear at 80 dB SPL. An earplug was deeply inserted in the left ear of the participant to minimize interference from other potential sound sources (e.g., audible vibrations of the DualSense Controller).

The presentation of both sensory stimuli followed a block paradigm (Figure 3A). Within each block, 100 /*da*/ trials were presented in an alternating polarity (i.e., 50 positive and 50 negative polarity) at a rate of 10.9 Hz. In total 40 blocks were presented for a single session, equating to 40 × 100 = 4000 /*da*/ trials per session. Importantly, the inclusion of a vibrotactile block was randomized with probability 0.5. Hence, only 20 blocks involved the simultaneous presentation of both sensory modalities where temporal synchronization was accounted for by setting adequate latencies. That is, we ensured a simultaneous peripheral stimulation between both modalities. Besides the audio-tactile condition, the remaining 20 blocks comprise only the auditory condition. The random ordering of consecutive conditions aimed to minimize potential confounding factors such as the expectancy effect. After the completion of a single session, a brief intermission of ~15 seconds was placed before proceeding to the next session. Each participant was exposed to a total of 3 repetitions, equating to a cumulative amount of 4000 × 3 = 12000 /*da*/ trials.

### 2.4 Data collection

The FFR was collected with the Eclipse EP15 system (Interacoustics, Middelfart, Denmark) using disposable Ag/AgCl gel electrodes and the EPA preamplifier (Interacoustics, Middelfart, Denmark). The electrodes were applied in a vertical montage with the active, reference and ground electrode located at Cz (top head), A2 (right earlobe) and Fpz (forehead) respectively (Figure 3A). The electrode impedance was kept below 5 kΩ. The acquired signal was subsequently recorded at 48 kHz (sampling rate) with the Steinberg UR44C audio interface (Steinberg Media Technologies GmbH, Hamburg, Germany). This audio interface was thus employed for both the delivery of auditory stimuli and the recording of the FFR. This arrangement aided in facilitating precise alignment between stimulation and recording, coordinated through a custom made Python script. Furthermore, during the experiment the participant was instructed to sit relaxed on a comfortable chair while watching a mute movie of choice (e.g., Charlie Chaplin). This visual distractor controls for attention and aimed to minimize head movements of the participant while promoting relaxation. Moreover, since the FFR signal is on the order of nanovolts, the experiments took place in an anechoic chamber that was enclosed in a Faraday cage to reduce (electrical) noise (Supplementary Figure S1) as well as possible auditory distractors.

### 2.5 Data analysis

Initial processing of the FFR was identical to that of Krizman and Kraus (2019). Hence, individual FFRs for each participant were filtered offline from 100 to 2,000 Hz with a second order digital Butterworth bandpass filter (Virtanen et al., 2020). After filtering, all trials were averaged over a 75 ms window, starting 15.8 ms prior stimulus onset. The artifact rejection criterion for invalid trials (e.g., myogenic activity) was set at ±23.8 μV. The averaged response corresponding to each polarity was added together (i.e., $\frac{\text{negative polarity + positive polarity}}{\text{2}}$), thereby minimizing the stimulus artifact and the cochlear microphonic (Skoe and Kraus, 2010; Krizman and Kraus, 2019). This procedure was followed for both the audio and the audio-tactile condition.

The stereotyped peak landmarks and their distinct timing for the FFR to the short /*da*/ stimulus are well established in literature for the auditory modality (Skoe et al., 2015), and are termed “V”, “A”, “C”, “D”, “E”, “F”, “O” respectively (Figure 3B). Peak “V” signifies the positive amplitude deflection associated with the stimulus onset and occurs at ~6-8 ms. Peak “A” is a negative amplitude deflection directly following “V”. Peak “C” reflects the transition from the onset burst to the onset of voicing. Subsequently, the three peaks “D”, “E”, and “F” are all negative deflections related to the voicing of the speech sound and are spaced ~8 ms apart (i.e., period of the fundamental frequency). Lastly, “O” constitutes a negative amplitude deflection characterizing the sound offset response. For both the audio and audio-tactile condition, peaks were identified and labeled manually from the averaged response of the participant.

To investigate the neural frequency processing, we were specifically interested in the spectral encoding of the FFR. Therefore, a Fast Fourier Transform (FFT) was applied to the formant transition of the /*da*/ sound as suggested in the study of Krizman et al. (2019). This transition corresponded to the 19.5-44.2 ms period in the averaged window. Zero-padding was applied to increase the spectral resolution to at least 1 Hz, and a Hanning window was applied to minimize spectral leakage. The obtained spectral encoding was then analyzed to investigate the fundamental frequency (F0: 75–175 Hz), a neural correlate associated with pitch perception. Additionally, the harmonics of F0 were examined. These harmonics were categorized into two bins: lower and higher harmonic content. The lower harmonics contained the first formant (F1) and ranged from 175–750 Hz. The higher harmonics, termed high frequency (HF), represented the frequencies between the first formant and the midbrain phase locking limits (up to 1050 Hz). Accordingly, the magnitude corresponding to the average spectral energy of each frequency bin (i.e., F0, F1, HF) were computed for comparison. Normative data for the auditory modality of the spectral encoding is visualized in Figure 3B.

Subsequent data analysis followed a within-subject design. More specifically, the FFRs under both audio and audio-tactile conditions were compared within each participant. These intra-individual comparisons were then pooled across participants to facilitate the statistical analyses. Accordingly, motivated by the benefits of simulation methods, paired permutation tests were employed to assess statistical significance (Holt and Sullivan, 2023). Suppose we collect the random variable *Y* = $X_{\text{audio-tactile}}^{i}$ - $X_{\text{audio}}^{i}$ for each participant *i*, where *X*^*i*^ represents any type of FFR measure (e.g., the average spectral energy of F0). Under the null hypothesis of exchangeability, a test statistic distribution $\bar{Y}$ is created by randomly permuting the condition labels (i.e., audio-tactile and tactile) for each participant, 100.000 times. Subsequently, the p-value with alternative hypothesis *H*_*A*_: $\bar{y}$ > 0 (frequency domain analysis) or *H*_*A*_: $\bar{y}$ ≠ 0 (time domain analysis) was determined for the observed unpermuted data. In case of multiple testing, a Bonferroni correction was performed to account for the family-wise type I error rate.

### 2.6 DualSense validation

Following the protocol outlined by Farina (2000), the frequency response characteristic of the DualSense controller was determined by employing an exponential sine sweep signal:


(1) $$\begin{array}{l} \text{x(t) = sin}\left(\frac{2\pi \cdot {\text{f}}_{\text{1}}\cdot \text{T}}{\text{ln}\left(\frac{{\text{f}}_{\text{2}}}{{\text{f}}_{\text{1}}}\right)}\cdot \left({\text{e}}^{\frac{\text{t}}{\text{T}}\text{ln}\left(\frac{{\text{f}}_{\text{2}}}{{\text{f}}_{\text{1}}}\right)}-1\right)\right) \end{array}$$

with start frequency f_1_ = 10 Hz, stop frequency f_2_ = 1,050 Hz and duration T = 50 s. In accordance with updated recommendations by Farina (2007), this single very long sweep was further processed by applying a fade-in using a one-sided Hanning window of 0.1 s. Measurements were conducted in the same anechoic chamber as the FFR recordings, and a GY-61 ADXL335 analog 3-axis accelerometer (Analog Devices inc, Wilmington, United States) was placed at both the left and right hand side of the controller to record the vibrations (Supplementary Figure S2A). Furthermore, to simulate the natural dampening effect while holding the controller, it was placed on top of an ordinary blanket. The analog signal of the accelerometer was captured using the Steinberg UR44C audio interface (Steinberg Media Technologies GmbH, Hamburg, Germany) at a sampling rate of 48 kHz.

Additionally, the controller's ability to handle the high stimulus rate of the */da/* trials was evaluated. This involved stimulating the controller with 4000 */da/* trials at a rate of 10.9 Hz (positive polarity only). The positions of the accelerometers, the recording apparatus, and the sampling rate remained unchanged compared to the acquisition of the frequency response. The response to each */da/* trial was then analyzed by comparing the root-mean-squared (RMS) value during a prestimulus period of 24 ms before onset, to the stimulus period of 40 ms after onset (after correcting the vibrotactile delay, see Supplementary Figure S2B). Subsequently, the signal-to-noise ratio (SNR) for each trial was computed according to:


(2) $$\begin{array}{l} \text{SNR}=\text{20}{\text{log}}_{\text{10}}\left(\frac{{\text{RMS}}_{\text{stimulus}}}{{\text{RMS}}_{\text{prestimulus}}}\right) \end{array}$$

For statistical analysis a similar paired permutation test was applied as detailed in section 2.5, now adapted to evaluate *Y* = $\text{20}{\text{log}}_{\text{10}}\left(\frac{{\text{RMS}}_{\text{stimulus}}^{i}}{{\text{RMS}}_{\text{prestimulus}}^{i}}\right)$ for each /*da*/ trial *i*. The *p*-values were determined following the alternative hypothesis *H*_*A*_: $\bar{y}$ > 0.

## 3 Results

This section outlines the results following the methods described in Section 2. Initially, it discusses the preliminary validation of the vibrotactile controller which demonstrated positive results supporting its suitability for the FFR recordings. Following parts of this section will focus on the time domain and frequency domain analyses of the acquired FFRs.

### 3.1 Preliminary validation of the vibrotactile controller

**Figure 4:**
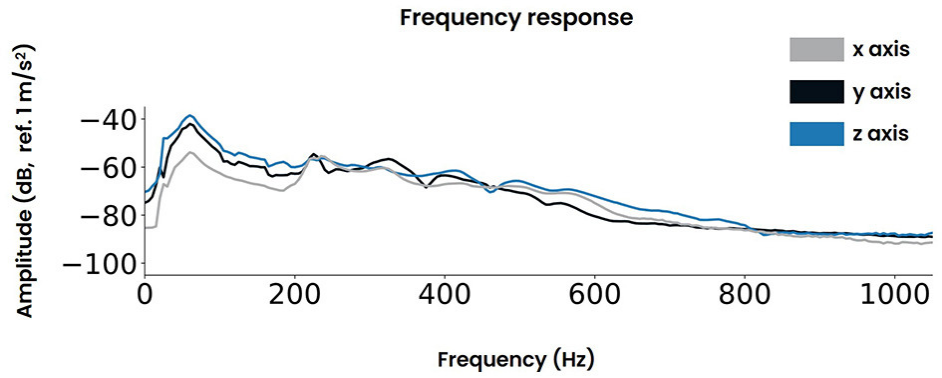
The frequency response of the vibrotactile controller for the three axes x, y, and z respectively.

The controller's frequency response was measured at two symmetrical locations adjacent to the vibrotactile actuators and the regions where the participant's hands make contact (Supplementary Figure S2A). Both locations produced similar responses as shown in Supplementary Figure S2C. The data obtained across both sides were averaged for each dimension (i.e., x, y, z) and are presented in Figure 4.

Furthermore, given the high stimulus rate of the transient */da/*, a valid concern arises regarding the inertia of the vibrotactile actuator. That is, if the actuator fails to return to its baseline level in time between two successive trials, the participant may not be able to differentiate between the individual trial instances. For vibrotactile stimuli presented to the hand, literature has shown that the minimum detectable separation between two successive stimuli is on the order of 8-12 ms (Merchel and Altinsoy, 2020). Those thresholds were found for noise and clicks at sensation levels of about 35 dB and for sinusoids with sensation levels of about 20 dB. While these stimulus parameters are distinct from the */da/* signal employed in this study, they do provide a reference point for estimating the temporal discrimination threshold of the vibrotactile modality. In this study, a wide seperation margin of 24 ms was employed. Specifically, a prestimulus period of 24 ms was defined as the baseline activity. Accordingly, the SNR was computed for 4000 consecutive */da/* trials, presented at 10.9 Hz. The averaged vibrotactile signals are visualized in Supplementary Figure S2B and the statistical results are summarized in Table 1. Hence, for each dimension the *p*-value neared 0 and large SNR values were found. Together, this suggested that the signal returned sufficiently to baseline levels between consecutive trials. The latencies observed for the onset of vibrations (Supplementary Figure S2B) were the result of the inertia of the vibrotactile actuator. This delay amounted to ~8 ms, a duration which is well below the perceivable threshold of ~40 ms (Brahimaj et al., 2023).

**Table 1:** Statistical results of the validation of the vibrotactile controller.

**Dimension**	***M***	***SD***	***p***	**Cohen's *d***
x	15.49	0.68	<0.001	22.83
y	14.74	1.56	<0.001	9.45
z	13.39	1.30	<0.001	10.25

For all trials (*n* = 4,000), the signal-to-noise ratio (SNR) is displayed for each dimension (i.e., x, y, and z) according to section 2.6. A significance level, α, of 0.05/3 = 0.016 is determined after Bonferroni correction.

The next step was to systematically calibrate the amplitude levels. Therefore, the peak-to-peak level was defined at the most dominant axis, orientated parallel to the built-in voice coil actuators of the controller (i.e., the z-axis). Accordingly, the 40-ms */da/* stimulus was calibrated to maintain a consistent amplitude of 0.85 m/s^2^ (Supplementary Figure S2B) for all remaining experiments.

### 3.2 FFR time domain

**Figure 5:**
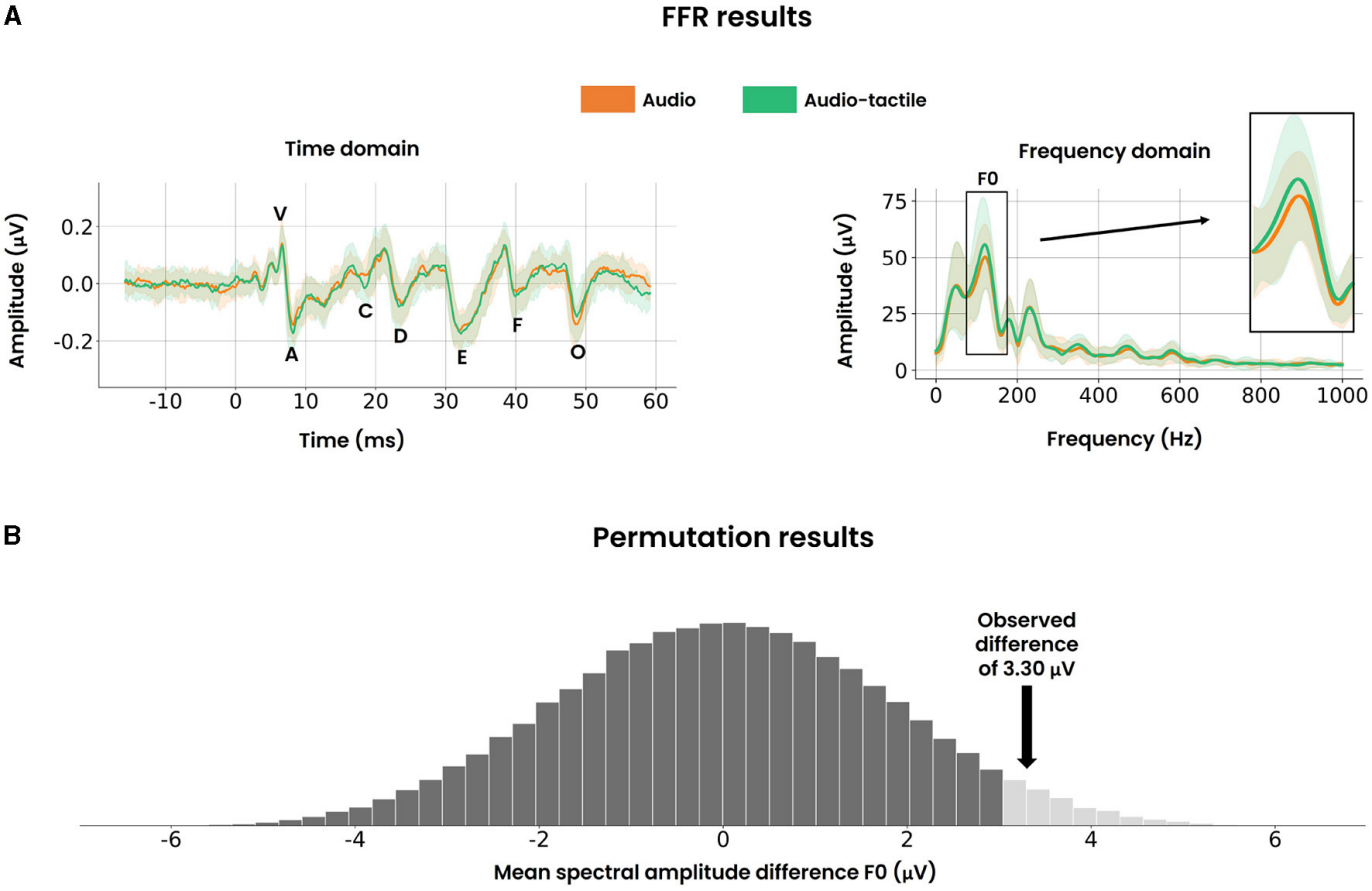
FFR results and permutation results. **(A)** Time domain representation (left) of the FFR where the added polarity was averaged across all participants. The mean value (solid curve) and standard deviation (fill areas around the mean) are displayed. The marked peaks follow the same definitions as detailed in Figure 3B. Spectral encoding of the time domain (right) for the window [19.5 ms–44.2 ms], averaged across all participants. Again, the mean value (solid curve) and standard deviation (fill areas around the mean) are displayed. The fundamental frequency bin (F0: 75 Hz–175 Hz) is highlighted on the right. **(B)** The null distribution of the mean spectral amplitude difference for F0, approximated by randomly permuting the condition labels. The light hued tail on the right represents the one-sided critical region for a significance level of 0.05. The black arrow demonstrates that the observed difference of 3.30 μV resides within this critical region, thereby providing statistical evidence to reject the null hypothesis.

The aim of the FFR time domain analysis was to determine whether the incorporation of the vibrotactile modality exerted any substantial effect on the timing of peaks, which could in turn confound the results of spectral analysis. A summary of the peak picking is presented in Table 2. No significant effect was found for all of the peak timing differences. The grand average across all participants is visualized in Figure 5A. Furthermore, the FFR onset delay of ~6-7 ms was in accordance with the neural transmission time (Skoe et al., 2015; Krizman and Kraus, 2019).

**Table 2:** Statistical results for the FFR time domain analysis.

**Landmark**	***M***	***SD***	***p***	**Cohen's *d***
V	−0.07	0.16	0.031	0.42
A	0.06	0.17	0.136	0.34
C	−0.21	0.71	0.154	0.30
D	−0.03	0.69	0.878	0.04
E	0.07	0.48	0.502	0.15
F	0.07	0.75	0.703	0.09
O	0.21	0.45	0.016	0.48

The letters V, A, C, D, E, F, and O refer to the distinct landmarks (Figure 3B). The peak timing difference of a landmark is displayed in ms and determined for all participants (*n* = 22). Negative values mean a later peak timing for the audio modality (audio > audio-tactile). A significance level, α, of 0.05/7 = 0.007 is determined after Bonferroni correction.

Additionally, control experiments were performed among 3 of the participants under vibrotactile stimulation only. That is, the procedure for recording the FFRs remained nearly identical, only now the insert earphone transducer in the right ear was replaced by an earplug. This modification facilitated a direct comparison between the vibrotactile condition and a baseline condition absent of both vibrotactile and auditory stimulation (Supplementary Figure S3). As a result, the vibrotactile modality only did not replicate the same waveform patterns as observed in Figure 5A. Instead, the response rather converges to the baseline level, suggesting that exclusively employing the vibrotactile signal in this study was insufficient to produce the stereotypical FFR.

### 3.3 FFR frequency domain

To assess the potential facilitatory role of vibrotactile stimuli in subcortical phase locking activity, it was crucial to examine if such an effect was reflected in the spectral encoding of the FFR. The grand spectral average across all participants is visualized in Figure 5A. The magnitude of the fundamental frequency (i.e., F0: 75 Hz–175 Hz) required special attention due to its correlation with pitch perception in earlier FFR studies. Focusing on F0, a paired permutation test was performed and visualized in Figure 5B. A significant difference of 3.30 ± 8.41 μV (*p*-value = 0.033, Cohen's *d* = 0.39, *n* = 22) was found, demonstrating enhanced phase locking activity of the fundamental frequency. In contrast, brief inspection of the higher harmonics F1 (175 Hz–750 Hz) and HF (750 Hz–1,050 Hz) revealed similar spectral amplitudes between both audio and audio-tactile conditions.

Additionally, spectral encoding of the control experiments under exclusive vibrotactile stimulation (Supplementary Figure S3) rendered similar amplitude levels as baseline activities. This restates that vibrotactile stimuli in isolation do not produce the observed FFR response.

## 4 Discussion

This study evaluated the hypothesis that vibrotactile stimuli enhance auditory phase locking in subcortical regions. The obtained FFRs from the audio-tactile condition corroborated this proposition, exhibiting increased neural synchronization at the fundamental frequency F0 specifically. Vibrotactile stimulation in the absence of sound produced a response that was indistinguishable from baseline levels. The latter hints that the observed multisensory effect does not arise from a mere linear summation but rather involves a super-additive interaction. Though, additional control experiments with statistical reporting should provide direct evidence to support this statement.

Previous research has already documented the role of the midbrain's inferior colliculus (i.e., the main neural source of the FFR) as a central processing hub for the integration of multisensory signals (Lohse et al., 2022; Kraus, 2021). This includes both feedforward projections (Jain and Shore, 2006) and corticofugal projections (Lohse et al., 2021) mediated by somatosensory substrates. As mentioned, the temporal coding mechanism in the sensory peripheries of both auditory and vibrotactile modalities are highly similar. It therefore appears plausible that the observed multisensory effect is facilitated through connections in the afferent pathway, exhibiting Hebbian learning. However, the potential contributions of efferent projections from both primary and non-primary cortical areas to the inferior colliculus cannot be discounted. For instance, an analogous study on the impact of visual stimulation on the auditory FFR similarly reported an increased representation of F0 (Musacchia et al., 2007). As mentioned by the authors, one explanation could be derived from the reverse hierarchy theory (Ahissar and Hochstein, 2004). This states that peripheral plasticity can be mediated by top-down corticofugal influences that originate from multisensory training. Likewise, Lakatos et al. demonstrated how a salient nonauditory stimulus (e.g., vibrotactile or visual) can enhance the neural excitability in the auditory cortex by phase resetting the ongoing oscillatory activity (Lakatos et al., 2009). This observation has been linked to the phenomenon of increased perceived loudness of auditory signals under concurrent vibrotactile stimulation (Lakatos et al., 2007). Furthermore, our research does not exclude the possibility that the observed effect may be attributed to a generic “novelty effect” rather than being an exclusive integration of audio-tactile stimuli. Hence, what mechanism exactly applies to the increased phase locking effect observed in the current study remains to be answered.

Our investigation did not detect enhanced phase locking at harmonic frequencies beyond the fundamental frequency. The harmonic content of acoustical stimuli has been linked to the perception of auditory timbre, which distinguishes the sounds of different instruments and voices (Saal et al., 2016). Analogously, the perception of tactile texture has been correlated with the harmonic content of skin vibrations (Manfredi et al., 2014). These harmonic frequencies are additionally reflected in the temporal coding of afferent neurons within both sensory peripheries. Hence, the substantial parallels between auditory timbre and tactile texture suggests a close relationship, similar to pitch processing. In this regard, previous research by Russo et al. has shown that auditory timbre could be discerned solely through vibrotactile stimulation (Russo et al., 2012; Russo, 2020). Yet, similar observations were not directly reflected in our findings. This may be attributed to both biological and technological constraints inherent in the present study. First is the limiting nature of perceived vibrations through the hand, exhibiting a relatively small bandwidth with optimal sensitivity around 240 Hz (Figure 1A). This sensitivity markedly declines at frequencies extending up to 1,000 Hz. As a result, the harmonic content of the employed */da/* stimulus might not have been effectively transmitted. Additional low-pass filtering imposed by the vibrotactile controller may have further exacerbated this issue. Specifically, its frequency response peaks around the fundamental frequency of */da/* and decreases for higher frequencies. Furthermore, the choice of adding both polarities favors the FFR to the temporal envelope which contains the low frequency content including the fundamental frequency (Krizman and Kraus, 2019). This is due to the fact that the temporal envelope is relatively phase invariant, thereby showing similar responses to both opposing polarities. Conversely, the temporal fine structure which includes the harmonic content is sensitive to the phase and thus cancels out when adding both polarities. Collectively, our design may have been biased toward the fundamental frequency, potentially obscuring any audio-tactile phase locking effects at higher harmonics. One improvement for future endeavors could be to explore the subtracted polarity since it accentuates the spectral fine structure at the expense of introducing more noise (Skoe and Kraus, 2010; Krizman and Kraus, 2019).

Besides, both the auditory and vibrotactile */da/* stimulus were presented at levels well above the detection threshold. It is, however, widely documented that multisensory interactions are most effective when employing stimuli that, in isolation, are minimally effective in producing neural responses. This relationship between the intensity of unisensory stimuli and the relative strength of the combined multisensory response is denoted as the principle of inverse effectiveness (Meredith and Stein, 1983). For example, Fu et al. demonstrated that tactile input fluctuating in-phase with auditory noise amplifies the fluctuations of the noise (Fu and Riecke, 2023). Importantly, this multisensory effect was largest when the auditory stimulus was weakest. Hence, it would be interesting to investigate whether the enhanced audio-tactile phase locking effect obeys the same rule. Future research should explore the use of weaker auditory stimuli to determine whether subsequent incorporation of vibrotactile stimuli could yield more pronounced effects with larger effect sizes.

Furthermore, despite utilizing speech stimuli and an ergonomic controller, the ecological validity of this study may still be questioned. The necessity for the high repetitiveness of a single stimulus to record the FFR is not typical in real-life scenarios. Additionally, correlations with behavioral measures have not been performed. Previous studies on the FFR has demonstrated a connection between enhanced neural synchronization at the fundamental frequency and improved pitch discrimination capabilities (Krishnan et al., 2005, 2010; Carcagno and Plack, 2011). It is therefore crucial to correlate the observed neurological effect presented in this study with behavioral outcomes to validate their functional significance. Accordingly, we propose that future studies should include a set of vibrotactile stimuli varying in specific parameters. For instance, it would be intriguing to explore vibrations with shifted pitches (i.e., shifted F0) compared to the auditory modality. Research by Yau et al. has indicated that the perception of auditory pitch shifts toward a concurrent vibrotactile pitch when the frequencies of both modalities are closely aligned (Yau et al., 2010). Investigating whether a similar pattern is reflected in the encoding of F0 in the FFR would be valuable. It would also be worthwhile to examine whether audio-tactile effects are restricted to vibrotactile stimulation of the hand only, or if other locations exhibit similar phenomena. Thus, future research should also consider varying the stimulation sites to assess its impact.

Additionally, the inclusion criteria for the participants in this study simply considered individuals with no self-reported hearing impairments. The latter statement could be strengthened by measuring actual audiograms. It would further be beneficial to gather a more comprehensive range of demographic data. Such data would enable the comparison of different populations to identify potential confounding variables that may influence the effectiveness of integrating the vibrotactile modality. An initial area of interest could be the role of musicianship. Musicians have shown to exhibit superior auditory processing abilities compared to non-musicians, including enhanced FFRs (Kraus, 2021). For example, the enhanced F0 representation in the audio-visual paradigm was more pronounced among musicians (Musacchia et al., 2007). Given that they also exhibit improved tactile frequency discrimination capabilities (Sharp et al., 2019), it would be logical to hypothesize a similar positive correlation between musicianship and the effectiveness of audio-tactile stimulation on the FFR. Additionally, extending this research to clinical populations, particularly individuals with hearing impairments such as cochlear implant users, offers an interesting avenue for research explorations. Little is understood regarding the neural plasticity occurring within this population, and empirical observations revealed distinct patterns of musical engagement compared to normal hearing people who more dominantly rely on the hearing senses (Fulford et al., 2011). Possibly, inclusion of vibrotactile stimuli in the study of the FFR may also exhibit enhanced effectiveness in such individuals.

In conclusion, using speech stimuli, our data show elevated phase locking activity at the fundamental frequency in human subjects under audio-tactile stimulation. Given that prior research has linked enhanced F0 encoding with augmented pitch processing capabilities, these findings hold promising practical implications. For example, implementing audio-tactile training to enhance pitch intelligibility may offer a practical approach for individuals struggling with pitch deficits, such as those with tone deafness or cochlear implant users. While this study represents an initial step toward exploring the potential benefits of audio-tactile stimulation, future studies are essential to further investigate its efficacy.

## Data Availability

The raw data supporting the conclusions of this article will be made available by the authors, without undue reservation.
