# Supplementary material for: Enhanced neural phase locking through audio-tactile stimulation
Source: Front Neurosci. 2024 Oct 2;18:1425398. doi: 10.3389/fnins.2024.1425398 (PMC11480033; doi:10.3389/fnins.2024.1425398)
Supplement: Supplementary file 1 [file Data_Sheet_1.pdf]

Supplementary Material

This document contains:

Supplementary Figures: S1, S2, S3

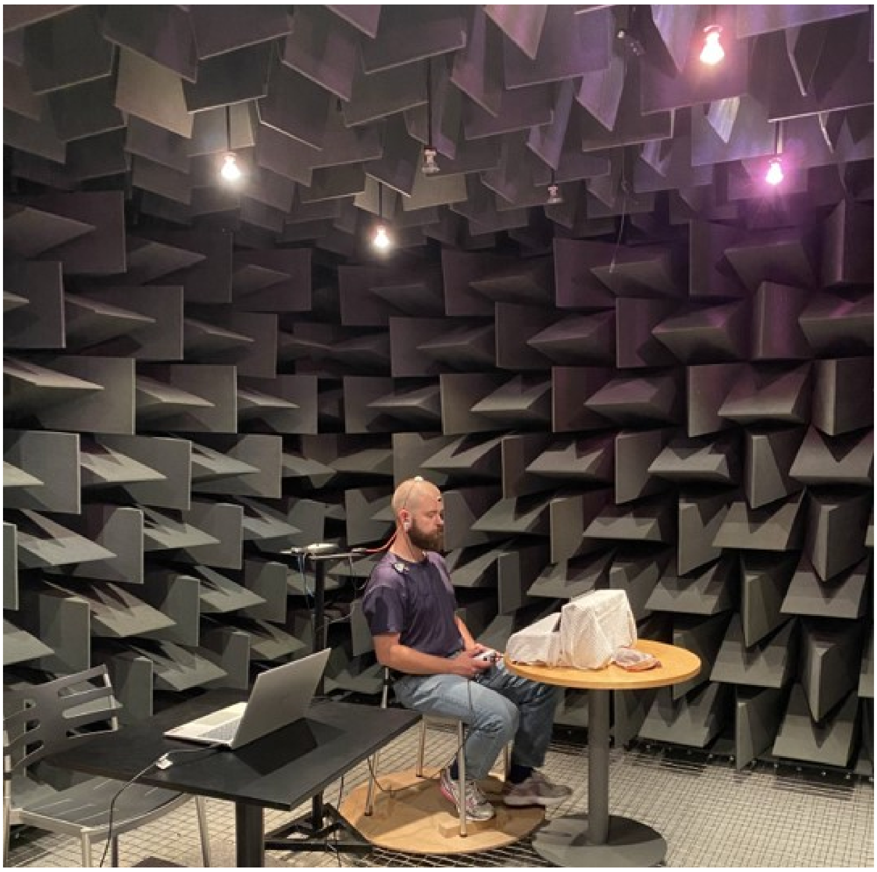

Figure S1. Setup for the FFR acquisition. The participant sits on a chair while watching a mute movie in the anechoic chamber that is enclosed in a Faraday cage.

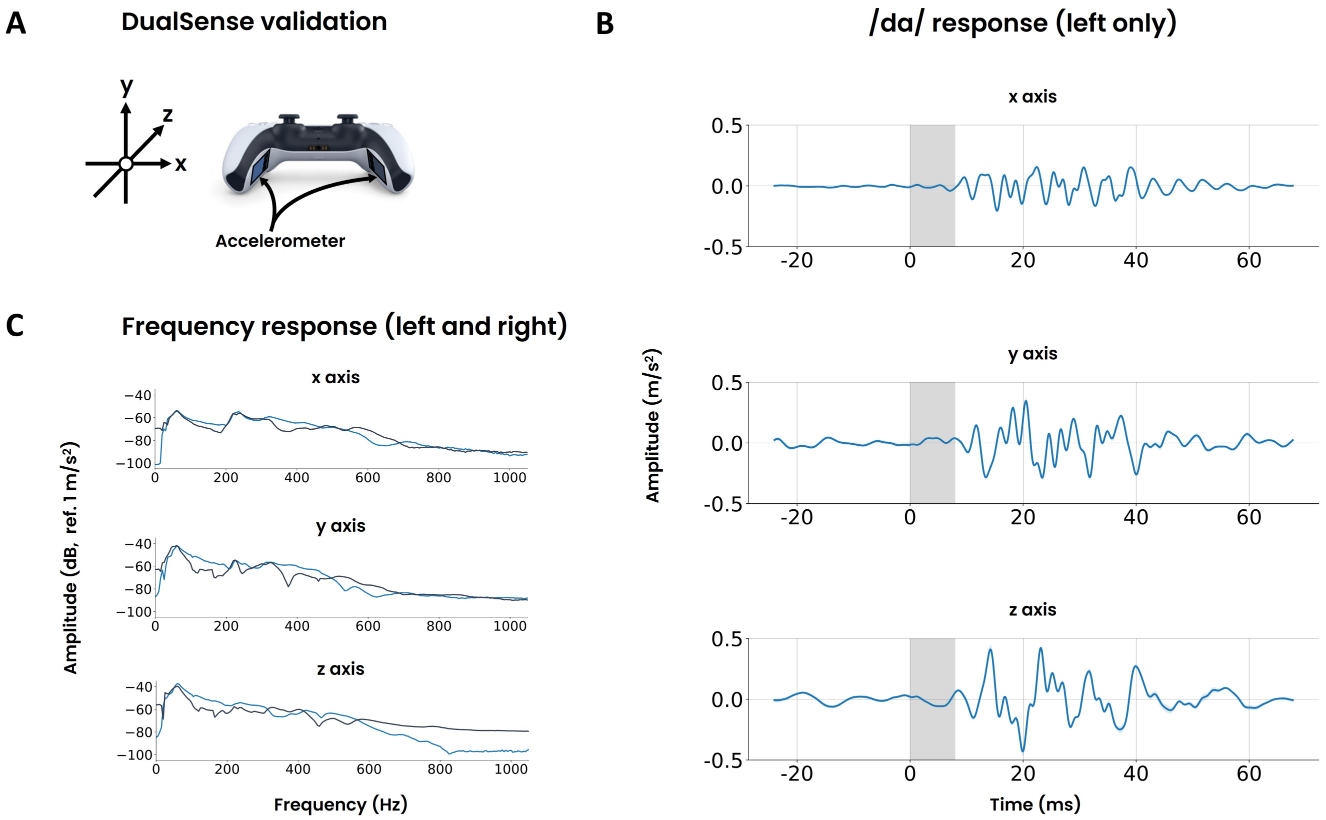

Figure S2. Setup for measuring the vibrotactile signal generated by the DualSense controller. **(A)** Schematic depiction of the 2 locations (i.e., left and right hand side) where the accelerometer was placed, along with the orientation of the 3 axis. **(B)** The solid line displays the averaged response to the 40-ms /*da*/ stimulus for all 3 axis measured by the left (light hued) accelerometer only. The fill areas around the means, though marginal, displays the standard deviation. The magnitude of the response, determined by the peak-to-peak value, is largest for the z axis (i.e., 0.85 m/s^2) which is used for calibration. The grey area marks the 8 ms delay compared to time = 0 corresponding to the 40-ms /*da*/ stimulus onset. **(C)** The frequency response for all 3 axis measured by the left (light hued) and the right (dark hued) accelerometer respectively.

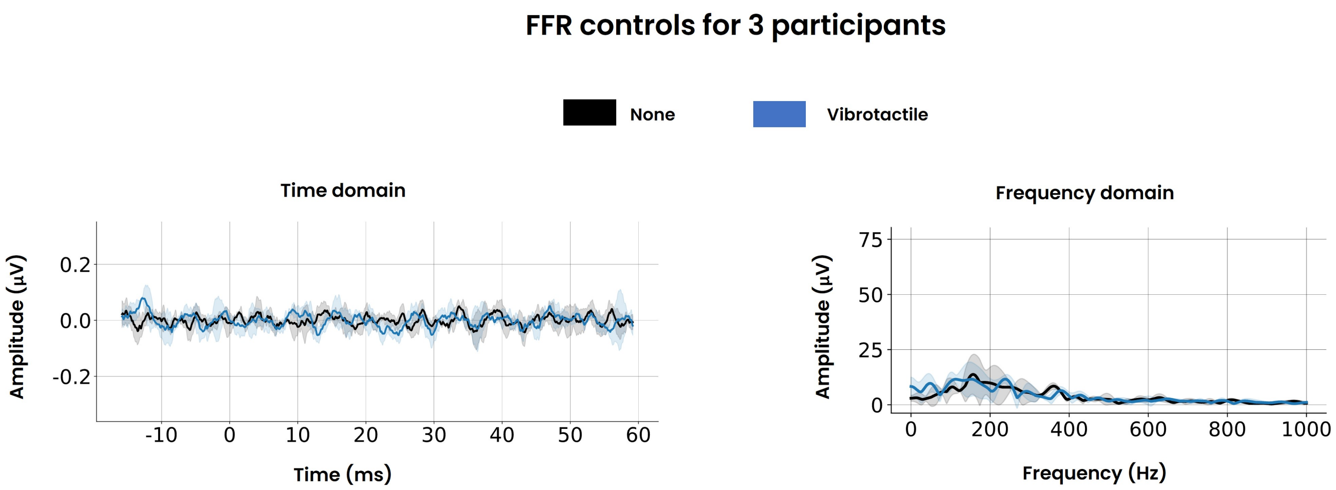

Figure S3. Control FFRs collected among 3 participants with earplugs inserted in both ears. Accordingly, the vibrotactile condition was compared to the baseline condition devoid of both vibrotactile and auditory stimulation. For the time domain and frequency domain representation, the mean value (solid curve) and standard deviation (fill areas around the mean) are displayed.
